# The Role of Eph/Ephrin-Driven Actomyosin Contractility in Promoting Cell Segregation and Boundary Formation

**DOI:** 10.3390/ijms27073250

**Published:** 2026-04-03

**Authors:** Jiaying Shi, Chunyu Lyu

**Affiliations:** School of Nursing, Zhejiang Chinese Medical University, 548 Binwen Road, Hangzhou 310053, China

**Keywords:** Eph receptor, ephrin ligands, actomyosin contractility, cell segregation, boundary formation

## Abstract

During development, tissues or organs are organized into distinct cell populations that do not intermix. The precise spatiotemporal arrangement of these populations establishes tissue boundaries and ensures proper morphogenesis. Signaling between membrane-bound Eph receptors and their ephrin ligands underlies the formation of multiple developmental boundaries, including those between germ layers, rhombomeres, and eye fields. Moreover, accumulating evidence indicates that actomyosin contractility serves as an important mechanical driver of Eph/ephrin-cell segregation and boundary formation. However, the mechanism by which Eph/ephrin signaling regulates actomyosin contractility have received relatively limited attention in previous reviews, particularly in the context of boundary sharpening. In this review, we focus on the interplay between Eph/ephrin signaling and actomyosin contractility and discuss how this interaction contributes to cell segregation and boundary formation.

## 1. Introduction

During early development, the embryo is subdivided into distinct regions or compartments that ultimately give rise to well-organized tissues and organs. Each region is physically demarcated by boundaries that prevent intermixing between adjacent cell populations [1]. These embryonic boundaries are essential for proper tissue separation and morphogenesis. Three major hypotheses have been proposed to explain the cellular mechanisms underlying: cell segregation and boundary formation differential adhesion, cell–cell repulsion, and differential interfacial tension [1,2,3,4]. Among these, the differential interfacial tension hypothesis (DITH), proposed by G. Wayne Brodland, suggests that both differential adhesion and differential cortical tension generated by actomyosin contractility contribute to variations in the stability of cell–cell contacts ultimately leading to cell segregation [5]. This process may also involve the coordinated action of multiple signaling pathways that limit cell mixing and contribute to boundary sharpening during normal embryonic development [6].

Eph receptor tyrosine kinase and their ephrin ligands constitute a critical signaling pathway during early embryonic development and play key roles in boundary formation [7]. Their expression patterns are highly complementary and widely distributed in early vertebrate embryos [8]. Due to the membrane-bound nature, Eph receptor and ephrin ligands primarily interact at interfaces between adjacent cells, thereby initiating bidirectional signals [9]. Such interactions have been shown to prevent inappropriate cell recognition and intermingling between distinct cell populations or tissues [10]. Importantly, Eph/ephrin signaling can regulate cell separation and border sharpening in vertebrates [11].

Actomyosin networks, composed of actin filaments and non-muscle myosin-II, are essential for generating contractile forces [12]. In various organisms and boundary systems, actomyosin enrichment has been consistently observed at tissue interfaces [13]. Under physiological conditions, these contractile forces contribute to changes in cell shape and play important roles in tissue patterning and morphogenesis [14,15]. Recent studies further indicate that Eph/ephrin signaling can promote actomyosin contractility, thereby causing cell segregation and boundary formation [11,16,17]. However, the extent to which Eph/ephrin bidirectional signaling differentially regulates actomyosin contractility during these processes has received relatively limited attention in previous reviews. Here, we summarize the general features of Eph/ephrin-mediated regulation of actomyosin contractility in cell segregation and boundary formation, including contexts such as germ layers, rhombomeres, and eye field. This review aims to provide a clearer understanding of the role of Eph/ephrin signaling in early development and to offer insights that may contribute to a deeper understanding of development disorders.

## 2. Eph Receptor and Its Ligand Ephrin

Eph receptors, the largest subfamily of receptor tyrosine kinases (RTKs), play vital roles in tissue patterning and morphogenesis through their interactions with cognate ephrin ligand [18,19]. Based on sequence homology and ligand-binding preferences, Eph receptors are classified into EphA and EphB subclasses [20]. In mammals, the EphA-subclass comprises nine members (EphA1-EphA8 and EphA10) in mammals, whereas the EphB subclass includes five members (EphB1-EphB4 and EphB6) [21].

Ephrins can similarly be divided into two classes, ephrin-A (ephrinA1–A5) and ephrin-B (ephrinB1–B3), according to their sequence conservation and binding affinities towards Eph receptors [21]. In general, EphA receptors preferentially bind to most ephrin-As, which are tethered to cell membrane via a glycosylphosphatidylinositol (GPI) anchor, whereas EphB receptors interact with ephrin-Bs ligands, which are possess a transmembrane domain and a short cytoplasmic domain [21,22]. However, a few exceptions exist; for example, EphA4 and EphB2 can interact with specific members of both ephrinA and ephrinB [22,23].

## 3. Eph-Ephrin Bidirectional Signaling

Like other RTKs, Eph receptors are type I transmembrane receptor tyrosine protein. Their extracellular domain includes a highly conserved N-terminal ligand-binding domain (LBD), followed by a cysteine-rich domain (CRD) and two fibronectin repeats (FN3) [24]. LBD functions as a ligand recognition and binding site [25]. CRD may be involved in receptor–receptor interactions that regulate oligomerization, a condition commonly observed upon ligand binding [26]. While the function of FN3 still needs to be explored. Intracellularly, there is a regulatory juxtamembrane region (JM), followed by a kinase domain and a sterile α motif domain (SAM) and a C-terminal PDZ (an acronym from three proteins: PSD-95, Dlg1, and ZO-1) domain-binding motif (PDM) [27]. The JM regulates the intrinsic activity of the adjacent kinase domain, which in turn mediates tyrosine phosphorylation of downstream protein substrates [18,28]. The resulting SAM domain may modulate receptor dimerization or receptor oligomerization and clustering by interacting with other proteins [29]. All ephrin ligands possess an approximately 20KDa N-terminal Eph receptor-binding domain [30]. In addition, Ephrin-Bs have an extra short cytoplasmic region with a C-terminal PDZ domain-binding motif that mediates protein–protein interactions [31].

**Figure 1:**
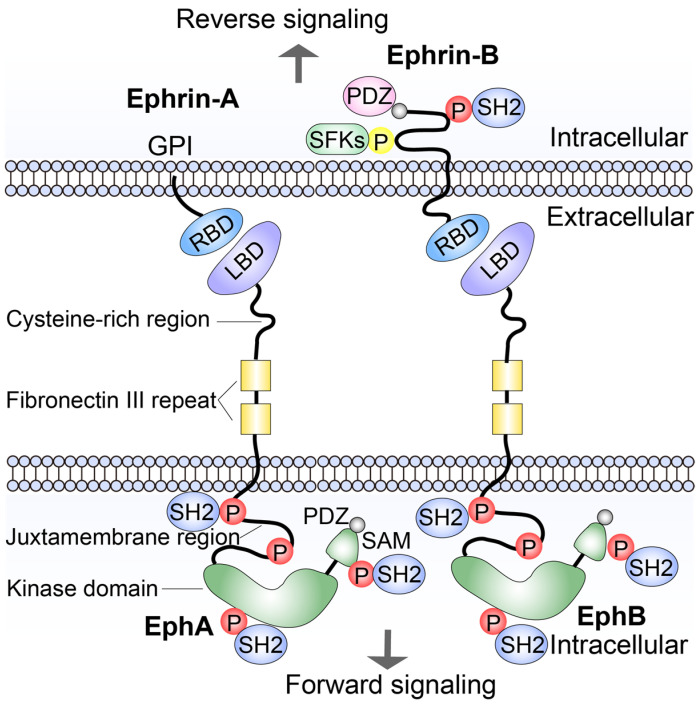
General features of Eph receptors and ephrin ligands. The receptor-binding domain (RBD) of ephrin-A and ephrin-B can interact with Eph receptors’ ligand-binding domain (LBD). SH2 and PDZ represent proteins containing these domains, SFKs are adaptor proteins, the red circle indicates tyrosine phosphorylation and the yellow circle indicates serine phosphorylation.

A distinguishing feature of Eph/ephrin signaling, compared with canonical RTK pathways, is its ability to mediate bidirectional signaling upon receptor–ligand interaction [32]. Since both Eph receptors and ephrins are transmembrane proteins, their interaction occurs at the interface of cell–cell contact [33]. Upon ephrin binding, Eph receptors initiate “forward signaling” in the receptor-bearing receptor-expressing cells through their intrinsic tyrosine kinase activity. Conversely, signals are also transmitted into ligand-expressing cells, a process referred to as “reverse signaling” [34]. The kinase domain of Eph receptors plays a crucial role in forward signaling. Ligand binding induces receptor dimerization and higher-order clustering, which promotes transphosphorylation and the activation of the receptor kinase domain [35]. Subsequently, two conserved tyrosines residues (JX1 and JX2) within the juxtamembrane region—major sites of autophosphorylation—become phosphorylated [36]. This phosphorylation relieves inhibitory intramolecular interactions with the receptor, thereby enabling full kinase activity [37]. These phosphorylated tyrosine residues serve as docking sites for downstream SH2 domain-containing proteins or adaptor proteins, including Nck and CrkII and Src family kinases (SFKs) [34,38,39]. Similarly, ephrinBs possess cytoplasmic domain containing multiple conserved tyrosine residues that can be phosphorylated. These residues provide binding sites to SH2 domains-containing proteins, such as SFKs, thereby initiating downstream reverse signaling pathways [40,41]. For example, tyrosine residue within the intracellular domain of ephrinB1 can be phosphorylated upon clustering with EphB2 [42,43]. In contrast, ephrin-As might transduce signals through alternative mechanisms. However, the mechanisms of the ephrin-As-mediated reverse signaling pathway remains incompletely understood and requires further investigation (Figure 1).

## 4. Regulation of Actomyosin Contractility by Eph/Ephrin Signaling

During development, tissue patterning and morphogenesis are dynamically regulated through changes in cell shape, which are primarily driven by the contractile actomyosin network [14,15,44]. Actomyosin contractility arises from the coordinated interaction between actin filaments and non-muscle myosin II, which slide antiparallel to generate contractile forces [45]. In addition, actin polymerization, network connectivity and turnover, as well as myosin-II dependent self-organization, further optimize the architecture of the actomyosin network and contribute to force generation [44,46].

**Figure 2:**
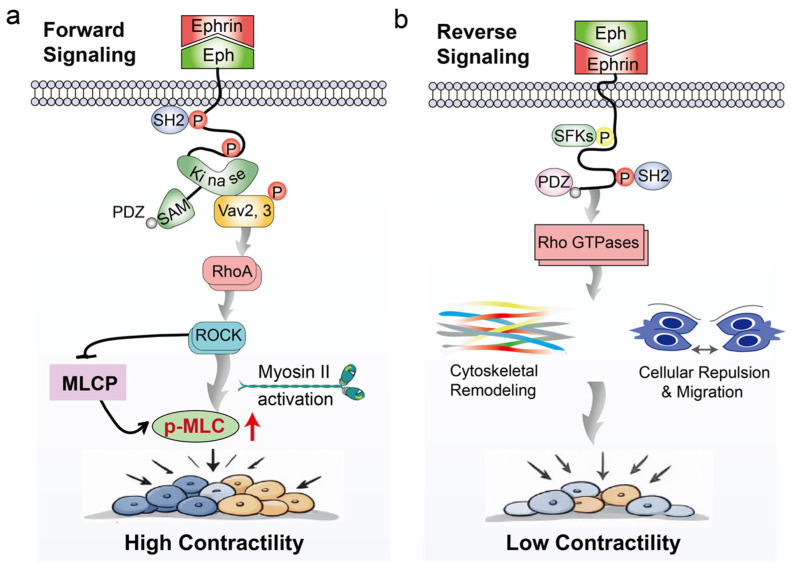
Differential regulation of actomyosin contractility by Eph/ephrin bidirectional signaling. (**a**) Eph receptor-mediated forward signaling activates the RhoA–ROCK–myosin II pathway, increasing MLC phosphorylation and actin polymerization, thereby enhancing cortical tension. (**b**) Ephrin-mediated reverse signaling regulates Rho GTPase activity and actin cytoskeletal remodeling through intracellular binding partners, contributing to cell repulsion and interfacial tension, but generating weaker contractile forces than forward signaling.

Eph/ephrin bidirectional signaling plays a critical role in modulating actomyosin contractility, with forward and reverse signaling exerting distinct effects. Forward signaling is primarily mediated by the intrinsic tyrosine kinase activity of Eph receptors. Autophosphorylation of receptor tyrosine residues generates docking sites for SH2 domain-containing proteins and guanine nucleotide exchange factors (GEFs, such as Vav2 and Vav3), which activate Rho family small GTPases, particularly RhoA [47]. Activated RhoA subsequently stimulates Rho-associated coiled-coil kinase (ROCK), enhancing myosin II activity through two complementary mechanisms, direct phosphorylation of myosin light chains (MLC) and inhibition of myosin light chain phosphatase (MLCP), thereby maintaining elevated levels of MLC phosphorylation. This promotes NMII motor activity and facilitates the assembly of cortical actomyosin structures [48]. Concurrently, RhoA can activate formin family proteins to promote actin polymerization, further stabilizing cortical actin filaments [49]. Collectively, Eph receptor-mediated forward signaling increases cortical tension in Eph-expressing cells, generating mechanical asymmetry at interfaces with neighboring ephrin-expressing cells. This asymmetry drives cell rounding, interface contraction, and the segregation of heterogeneous cell populations, ultimately contributing to boundary sharpening (Figure 2a). Pharmacological inhibition of ROCK or blockade of myosin II function significantly attenuates Eph/ephrin-induced contractility and cell segregation, highlighting the central role of the RhoA–ROCK–myosin II axis in forward signal-mediated regulation of cortical tension.

By contrast, reverse signaling depends on intracellular tyrosine phosphorylation sites and PDZ-binding motifs within ephrinB, which recruit SH2- and PDZ domain-containing proteins to regulate Rho GTPase activity, actin cytoskeletal remodeling, and cell migration [50]. Although reverse signaling can contribute to interfacial tension regulation and cell repulsion, it nearly could not generate contractile forces compared with forward signaling (Figure 2b). Taken together, Eph/ephrin signaling regulates cortical actomyosin contractility, interfacial tension, and cell sorting primarily through Eph receptor-mediated activation of the Vav–RhoA–ROCK–myosin II pathway, while ephrin reverse signaling provides additional modulatory input to fine-tune cytoskeletal dynamics and tissue mechanical properties.

## 5. The Role of Eph/Ephrin in Cell Segregation and Boundaries Formation

Eph receptors and ephrin ligands play crucial roles in the segregation of distinct cellular populations and the formation of sharp tissue boundaries [7]. As transmembrane proteins, they interact at sites of direct cell–cell contact [22]. Once interacting, bidirectional signaling is activated in both interacting cells, often leading to their retraction and migration in opposite directions [51,52]. This mechanism prevents the intermingling of heterotypic cells and provides a fundamental basis for Eph/ephrin-mediated cell separation and tissue boundary formation [10,11,53]. Moreover, numerous studies have demonstrated that Eph-ephrin signaling mediates cell separation and border sharpening by modulating diverse cellular behaviors in vertebrates, including cell polarization, migration, adhesion, repulsion, and cytoskeletal dynamics such as actomyosin contractility [11,13,54,55,56]. Importantly, actomyosin contractility itself could drive cellular segregation by promoting boundary formation [16]. For example, actomyosin-dependent cortical tension has been shown to drive germ-layer progenitor cell sorting during *Zebrafish* gastrulation [57]. Treatment of progenitor cells with blebbistatin, an inhibitor of myosin II activity, induces cell-cortex tension to comparable levels across different progenitor cell types [57]. Similarly, enrichment of actomyosin cytoskeletal components has been observed in early *Drosophila* embryo, suggesting that actomyosin-based barriers might restrict cell invasion into invading neighboring compartments [58]. Given these observations, an important question arises: could Eph/ephrin signaling promote cell sorting and boundary formation by regulating the contractility of actomyosin? In the following sections, we will focus on the mechanisms by which Eph/ephrin-driven actomyosin contractility regulates cell segregation and boundary formation in several developmental contexts, including germ layers during gastrulation, rhombomeres segmentation, and eye field segregation during forebrain morphogenesis.

### 5.1. Segregation of Germ Layers (Ectoderm–Mesoderm Boundary and Notochord–Presomitic Mesoderm) During Gastrulation

**Figure 3:**
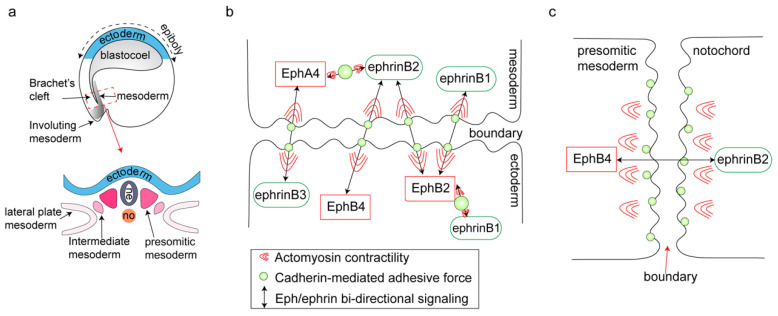
Segregation of germ layers during gastrulation. (**a**) The diagram of ectoderm–mesoderm boundary and notochord–presomitic (PSM) boundary in the *Xenopus*. (**b**) At the ectoderm–mesoderm boundary, heterotypic activation of Eph/ephrin signaling can generate strong actomyosin contractility to overcome the cadherin adhesive force, which leads to cell repulsion. Regarding contractility, weak homotypic activation of Eph/ephrin signaling cannot generate enough actomyosin contractility and antagonism increased cadherin adhesive forces. (**c**) Bidirectional EphB4/ephrinB2 signaling promotes actomyosin contractility and inhibits cadherin clustering to maintain the notochord–presomitic mesoderm boundary.

During early development, embryos transition from blastula stage and reorganize into a multilayered structure through a process known as gastrulation [59]. The formation of gastrula establish the primary germ layers, in which properly defined ectoderm–mesoderm and notochord–presomitic mesoderm (PSM) boundaries are essential for maintaining tissue integrity and preventing the intermixing of cells with distinct identities—an important prerequisite for normal morphogenesis [60] (Figure 3a). The gastrulation of *Xenopus* is a prototypical example of such a type of morphogenesis [61]. In *Xenopus* gastrula, mesodermal cells migrate along the inner surface of the ectoderm blastocoel roof (BCR), forming a distinct boundary between the two germ layers known as Brachet’s cleft. During this translocation, mesodermal cells can come into direct contact with ectodermal cells; however, intermixing is prevented, by Eph/ephrin signaling [62,63,64]. Notably, Eph receptor and ephrin ligands are differentially expressed in *Xenopus* gastrula embryos: EphB4, EphB2, ephrinB3, and ephrinB1 are predominantly expressed in the ectoderm, whereas EphA4, ephrinB2 and ephrinB1 are expressed in mesoderm [6,65]. Complementary expression patterns of ephrins and Eph receptors are enriched on opposing sides of the boundary, contributing to its maintenance [65] (Figure 3b).

Accumulating evidences indicates that Eph/ephrin signaling regulates cell segregation at the ectoderm–mesoderm boundary in *Xenopus* [64]. Both theoretical models and experimental evidence suggest that segregation at this interface depends on strong actomyosin contractility triggered by heterotypic cell contacts, which is required to overcome cadherin-mediated adhesion. In addition to Eph/ephrin signaling, cadherin-mediated adhesion could antagonize actomyosin contractility and thereby modulate cell repulsion. Effective tissue separation occurs only when repulsive forces mediated by Eph/ephrin signaling exceed adhesive forces generated by cadherins. However, because Eph/ephrin molecules and cadherins are expressed in overlapping patterns within both tissues, Eph/ephrin signaling between homotypic cells induces only weak actomyosin contractility. Consequently, the resulting repulsive forces are insufficient to counterbalance cadherin-mediated adhesion, thereby preventing separation between homotypic cells [66]. Collectively, these findings suggest that Eph/ephrin signaling locally regulates actomyosin contractility to generate differential cortical tension, promoting cell sorting at boundaries rather than intermixing.

As development proceeds, Brachet’s cleft further subdivides the dorsal mesoderm into three parts: the notochord is flanked by the PSM, also called paraxial mesoderm [7]. Similar to the ectoderm–mesoderm boundary, the formation of the notochord–PSM boundary is closely associated with Eph/ephrin signaling and actomyosin contractility. Cadherins mediate cell–cell adhesion through the formation of multimeric clusters. Initially, it was proposed that the disruption of cadherin clustering was the primary driver of boundary formation. However, Florence Fagotto and colleagues demonstrated that treatment with blebbistatin led to the accumulation of cadherin clusters at heterotypic contacts, resulting in increased cell mixing along the notochord–PSM boundary [11]. In this system, EphB4 and its ligand ephrinB2 exhibit complementary expression patterns: EphB4 is enriched in the presomitic mesoderm, whereas ephrinB2 is expressed in the notochord. Bidirectional EphB4/ephrinB2 signaling across the boundary enhances local contractility, promoting detachment of the plasma membrane from the actin cortex and inhibiting cadherin clustering (Figure 3c) [11]. Therefore, in addition to the role of actomyosin contractility in boundary separation, Eph/ephrin signaling itself is also crucial for the formation of the notochord–PSM boundary. In other words, Eph/ephrin-dependent actomyosin contractility is responsible for suppressing cadherin clustering and thereby promoting boundary formation.

### 5.2. Hindbrain Compartmentalization

**Figure 4:**
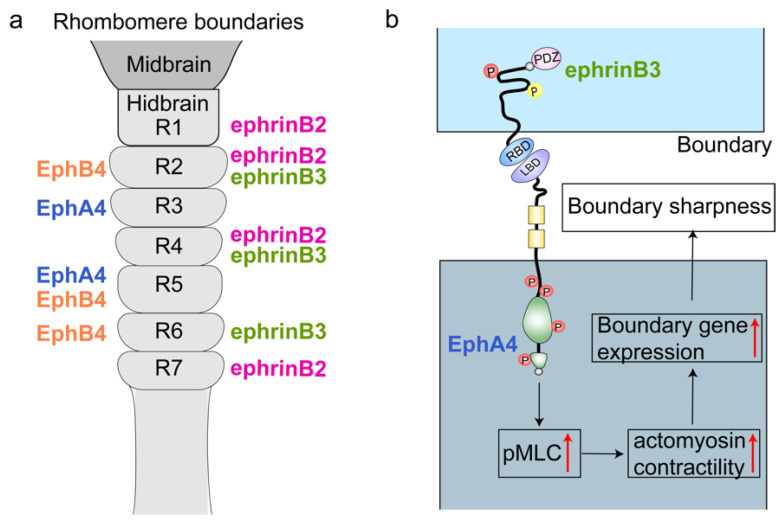
Model of Eph/ephrin signaling and hindbrain boundary compartmentalization. (**a**) The diagram of rhombomere boundaries and relevant expression pattern of Eph receptors and ephrin ligands. (**b**) During the process of segmentation of rhombomere, once the ephrinB3 ligand binds with the EphA4 receptor, the EphA4 forward signaling would increase the level of myosin light chain phosphorylation (pMLC) and actomyosin contractility, which then upregulate the expression of boundary gene and help sharp boundaries.

During early vertebrate development, the neural epithelium of the hindbrain is subdivided into a series of morphologically distinct segments termed rhombomeres (r1–r7), separated by sharp boundaries that prevent the intermixing of cells with distinct identities [67,68] (Figure 4a). Rhombomere segmentation is essential for establishing the structural organization of the hindbrain neuroepithelial and maintaining proper brain patterning [69]. However, these boundaries are initially diffuse [70,71,72]. Consistently, several Eph receptors and ephrins produce complementary expression patterns in the hindbrain [73]. In *Zebrafish*, EphA4 and EphB4 are expressed in rhombomeres r3/5 and r2/5/6 respectively, whereas their ligands ephrinB2 and ephrinB3 are expressed in r1/2/4/7 and r2/4/6, respectively [74,75].

Recent studies suggest that Eph/ephrin signaling in hindbrain compartmentalization may be closely linked to its regulation of actomyosin contractility. Activation of Eph receptors at segmental interfaces is frequently accompanied by increased p-MLC, a widely observed indicator of enhanced actomyosin contractility. The resulting local increase in cortical tension is thought to facilitate the segregation of adjacent cell populations and contribute to the progressive sharpening of rhombomeric boundaries [76]. However, although these observations support the hypothesis that actomyosin-mediated mechanical tension participates in boundary organization, the current evidence remains insufficient to directly demonstrate that changes in contractility represent the primary driving force of boundary formation.

Perturbation experiments targeting actomyosin function provide further, albeit still indirect, support for this connection. For example, treatments that increase myosin II phosphorylation have been reported to expand the expression domains of boundary-associated marker genes. Conversely, inhibition of myosin II activity or disruption of actin polymerization could affect the expression of these markers and disrupt boundary organization [13]. These findings suggest that actomyosin contractility may contribute to the stabilization or maintenance of hindbrain boundaries. Nevertheless, it remains unclear whether the observed increase in contractility actively drives cell segregation or instead arises as a downstream consequence of other segmentation mechanisms.

In this context, Eph receptor forward signaling appears to play a particularly prominent role in regulating cytoskeletal dynamics at segmental interfaces. Activation of EphA4 promotes p-MLC and increases cortical tension, a process that is also associated with nuclear translocation of the mechano-transduction effector Taz. Once in the nucleus, Taz can cooperate with the transcription factor Tead1a to regulate the expression of boundary-specific genes (Figure 4b) [76]. These findings suggest that mechanical signals generated by actomyosin activity may further influence transcriptional regulation in boundary cells.

### 5.3. Eye Field Segregation During Forebrain Morphogenesis

During forebrain morphogenesis, the formation of diencephalon, telencephalon, and eye field requires precise cell sorting to establish sharp tissue boundaries [77]. High-resolution live imaging in *Zebrafish* shows that presumptive eye cells first acquire apicobasal polarity and intercalate between marginal cells, during the bilateral expansion of the optic vesicles [78]. Initial evidence supporting the role of Eph/ephrin signaling in maintaining eye field organization includes two key observations. First, several Eph receptors and ephrins are expressed in complementary regions of the anterior neural plate (ANP) [79]. Second, overexpression of a truncated form of EphA4 disrupts the normal eye field organization, preventing cells in prospective optic vesicles from adjacent ANP regions [80]. Subsequent studies have focused on the role of Eph/ephrin signaling in forebrain morphogenesis, particularly at the eye field-telencephalic boundary. Consistent with mechanisms observed in rhombomeres and germ layer boundaries, the boundary at the margins of eye field is outlined [77], specifically, Eph receptors and corresponding ephrins at the interface between eye field and neighboring ANP domains. Upon Eph/ephrin activation, both F-actin and phosphorylated myosin II light chain accumulate at the eye field/telencephalon border. Those accumulated actomyosin cables might increase cortical tension and facilitate cell segregation and the formation of sharp boundaries within the ANP and at the eye field/telencephalon interface.

### 5.4. Cell Segregation

In addition to mediating boundary formation through the regulation of actomyosin contractility, Eph/ephrin signaling also functions at the cellular level to control cell segregation [81]. Cellular self-organization via cell segregation is a fundamental process underlying morphogenesis and boundary formation during development [82]. From a biophysical perspective, Eph/ephrin-driven cell segregation depends on actomyosin contractility [16]. When considered together with cell adhesion, the mechanism aligns with the differential interfacial tension hypothesis, which posits that cell segregation is governed by a dynamic balance between adhesion forces and cortical tension [5,57]. Cell adhesion is primarily mediated by cadherin, whereas Eph/ephrin signaling promotes actomyosin contractility, thereby generating cortical tension [16,83]. Recent studies further suggest that the forces driving cell separation arise from the mechanical coupling between cadherins and the contractile actomyosin cortex, which transmits intracellular tension to cell–cell interfaces [16]. Consequently, high interfacial tension destabilizes cell–cell contacts and promotes cell separation [16]. Importantly, the antagonism interplay between cadherin-mediated adhesion and actomyosin-generated tension is a key determinant of cell segregation [2]. Within cell co-expression of Eph receptors and ephrins, cadherin depletion decreases cohesion which cannot antagonize cortical tension generated by Eph/ephrin signaling and lead to homotypic cell repulsion [65,84]. While overexpressing cadherin in ephrin-expressing cells, increased homotypic adhesion antagonizes cortical tension, which cannot separate homotypic ephrin-expressing cells, but causes a greater different cortical tension compared with the neighboring heterotypic Eph-expressing cell interface and eventually leads to heterotypic cell segregation [85]. Importantly, cadherin-mediated adhesion and downstream actomyosin contraction of Eph-ephrin signaling might act oppositely to impact cell segregation.

In addition, recent studies by Abigail A Kindberg et al. have also elucidated the biomechanical mechanisms driving EPH/EPHRIN-based cell separation, in which differences in interfacial tension are regulated by actomyosin contractility controlling cell self-organization. Indeed, in normal conditions, in vitro cell–cell contact angle assays revealed a significantly decreased contact angle at the interface between heterotypic ephrinB1 and EphB2-expressing HEK293 cells, while homotypic ephrinB1:ephrinB1 and EphB2:EphB2 cell pairs come into close contact with each other. However, the contact connection between heterotypic cells is disrupted once Eph/ephrin signaling is blocked by exogenous unclustered ephrinB1-Fc. Meanwhile, Eph/ephrin signaling can increase cortical tension at the heterotypic cell interface to promote cellular segregation. Echoing this, cell detachment was almost completely abolished when the Eph-expressing cell and ephrin-expressing mixed cell population were treated with the actomyosin contraction inhibitor (Rho kinase (ROCK) inhibitor: Y27632 and myosin. Light chain kinase (MLCK) inhibitor: ML7). Furthermore, the heterotypic interfacial tension between EphB2 and ephrinB1 cell pairs would also diminish after blebbistatin treatment. Likewise, the increased stiffness of EphB2 cells and EphrinB1 cells subjected to cell isolation in mixed culture also indicated that Eph/Ephrin signaling increased actomyosin cortical tension during cell isolation [16]. Therefore, this high heterotypic tension primarily depends on actomyosin contraction downstream of Rho kinase (ROCK) and myosin light chain kinase (MLCK).

Not only in vitro cell experiments but in vivo chimeric mouse model experiments have also verified that the Eph/ephrin signaling pathway drives actomyosin contractility to regulate cell segregation [16,17]. For instance, the cell segregation happened in the anterior palate of *EphB1^loxXGFP/+^*; *Shox2^IresCre/+^* embryos (a *Shox2^IresCre/+^* allele can promote recombination and robust cell segregation in the anterior palate), whereas *EphB1^loxXGFP/+^*; *NMIIA^lox/lox^*; *NMIIBlox/lox*; *Shox2^IresCre/+^* embryos exhibited unsorted XGFP positive cells appearing throughout the palate (X-linked β-actin–GFP transgene (XGFP); a *Shox2^IresCre/+^* allele can promote recombination and robust cell segregation in the anterior palate; non-muscle myosin IIA (NMIIA) and non-muscle myosin IIB (NMIIB) allele can disrupt actomyosin contractility) [16]. In addition, mouse genetics experiments demonstrated that ephrin-B1 can drive cell segregation in the neuroepithelium progenitor population shortly after the onset of ephrinB1 expression [17]. The results of these in vivo experiments are highly consistent with the notion that EphB/ephrin signaling leads to cell sorting through Rho kinase-dependent generation of cortical actin differences between ephrin-B1 and EphB-expressing cells [17]. Taken together, actomyosin contractility is responsible for Eph/ephrin heterotypic interfacial tension and regulates heterotypic cell segregation in vitro and vivo.

## 6. Discussion

Tissue patterning has traditionally been attributed to three principal mechanisms: differential adhesion, cell–cell repulsion, and differential interfacial tension. In this review, we reconsider Eph/ephrin signaling from the perspective of actomyosin contractility. We emphasize that asymmetric cortical tension between neighboring cells may refine the differential interfacial tension model. Several developmental systems support this idea. In mesenchymal mesoderm cells, EphB/ephrin-B signaling induces actomyosin contractility. This contractility generates repulsive forces. These forces prevent mesenchymal mesoderm cells from invading the ectoderm. At the notochord–PSM border, Eph/ephrin signaling also enhances actomyosin contractility. This increase inhibits cadherin aggregation and forms a non-adhesive boundary. In the hindbrain neural epithelium, EphA4 signaling promotes actomyosin contractility. This activity generates tensile forces that sharpen rhombomere boundaries. At the eye field–telencephalon boundary, Eph/ephrin activation increases myosin II phosphorylation. This change likely produces tensile forces that support boundary formation. At the cellular level, Eph/ephrin signaling increases interfacial tension. It does so by enhancing actomyosin contractility and reducing stable cell–cell contacts. These effects promote cell segregation. In vivo studies support this mechanism. For example, chimeric mouse models show that actomyosin contractility is required for Eph/ephrin-mediated cell segregation. Taken together, Eph/ephrin signaling likely contributes to vertebrate boundary formation by regulating actomyosin contractility.

### 6.1. Relative Contributions of Forward and Reverse Eph/Ephrin Signaling to Contractility Regulation

Despite the accumulating evidence linking Eph/ephrin signaling to actomyosin contractility, an important unresolved issue is the relative contribution of Eph forward versus ephrin reverse signaling to the generation of contractile forces during boundary formation. Because Eph receptors and ephrin ligands are both membrane-bound molecules, their interaction typically induces bidirectional signaling in the contacting cells. In principle, either signaling direction could influence cytoskeletal organization and mechanical tension at heterotypic interfaces. However, current evidence suggests that the two signaling directions may contribute differently to the regulation of contractility.

Forward signaling initiated in Eph receptor-expressing cells appears to play a particularly prominent role in promoting actomyosin activation. For example, biochemical studies have shown that Eph receptor activation can stimulate Rho family GTPases through guanine nucleotide exchange factors, leading to activation of the RhoA–ROCK pathway and increased phosphorylation of myosin light chain [86]. Consistent with this mechanism, genetic and cell biological studies have demonstrated that Eph receptor activation can elevate cortical tension and drive cell repulsion at heterotypic interfaces. In particular, the work of O’Neill and colleagues demonstrated that kinase-dependent forward signaling is sufficient to generate a cortical actomyosin differential between Eph-expressing and ephrin-expressing cells, thereby promoting cell segregation [17]. These findings suggest that forward signaling can directly enhance actomyosin contractility within the Eph receptor-expressing population.

By contrast, ephrin-mediated reverse signaling is generally thought to modulate cytoskeletal organization through adaptor proteins containing SH2 or PDZ domains and through regulation of Rho family GTPases [87]. Although reverse signaling has been implicated in cell adhesion, migration, and cytoskeletal remodeling, its contribution to contractile force generation appears to be more context-dependent. In some developmental systems, reverse signaling may cooperate with forward signaling to reinforce cytoskeletal asymmetry across tissue interfaces, whereas in others it may primarily fine-tune cell behavior rather than directly generating contractile tension.

Importantly, the observation that actomyosin contractility frequently accompanies boundary formation does not necessarily imply that it represents the primary driving force of the process. Developing tissues are subjected to multiple mechanical influences, including differential adhesion, tissue-scale tension, cell proliferation, and morphogenetic movements [88]. In this context, increased actomyosin contractility at compartment boundaries could arise as a consequence of other patterning mechanisms rather than acting as the initiating driver. Therefore, although actomyosin-based tension clearly contributes to the stabilization and sharpening of boundaries, further studies are needed to determine whether it functions as the dominant mechanical force or instead operates together with additional biomechanical processes during tissue segregation.

### 6.2. Functional Redundancy Among Eph/Ephrin Family Members and Experimental Strategies to Dissect Their Roles

Another challenge in understanding Eph/ephrin-mediated regulation of actomyosin contractility is the considerable redundancy within the Eph receptor and ephrin ligand families. Multiple Eph receptors and ephrins are often expressed in overlapping or complementary patterns within the same developmental tissues, and many receptors are capable of interacting with more than one ligand [89]. As a consequence, genetic perturbation of a single Eph receptor or ephrin ligand may not fully reveal its functional contribution, because other family members can partially compensate for its loss. This redundancy complicates efforts to determine the specific Eph/ephrin pairs responsible for regulating contractility and boundary formation in vivo.

To address this issue, several experimental strategies could be employed. First, combinatorial genetic approaches, such as simultaneous knockdown or knockout of multiple Eph receptors or ephrin ligands, could help uncover redundant functions that are masked in single-gene perturbations [90]. Second, mosaic or chimeric models in which Eph- or ephrin-deficient cells are introduced into otherwise normal tissues may allow the analysis of cell-autonomous versus non-cell-autonomous contributions to interfacial tension and cell segregation [17]. Third, the use of engineered receptor variants that selectively disrupt forward or reverse signaling provides a powerful approach to dissect the relative contribution of each signaling direction to actomyosin regulation [91]. Finally, recent advances in quantitative imaging and biophysical measurements, including laser ablation, traction force microscopy, and tension sensor-based approaches, may enable more direct assessment of mechanical forces generated at Eph/ephrin-dependent interfaces. Together, these experimental strategies could provide important insights into how specific Eph/ephrin interactions contribute to the mechanical regulation of tissue boundaries.

### 6.3. Current Limitations in Understanding Eph/Ephrin-Mediated Mechanical Regulation of Tissue Boundaries

Despite significant progress in understanding the relationship between Eph/ephrin signaling and actomyosin contractility, several limitations remain in the current field. One major challenge lies in the difficulty of directly measuring mechanical forces within living embryos. While changes in myosin phosphorylation, actin organization, or cortical enrichment are often used as indicators of contractility, these measurements do not necessarily provide a direct quantification of mechanical tension. Consequently, distinguishing whether actomyosin contractility actively drives boundary formation or simply accompanies other morphogenetic processes remains challenging.

Another limitation concerns the redundancy and complexity of Eph/ephrin signaling networks. As discussed above, the overlapping expression patterns and promiscuous binding relationships among Eph receptors and ephrin ligands make it difficult to assign specific functions to individual receptor–ligand pairs. Moreover, Eph/ephrin signaling often interacts with other pathways that regulate cytoskeletal dynamics, including cadherin-mediated adhesion, Notch signaling, and mechano-transduction pathways. These interactions further complicate efforts to isolate the precise contribution of Eph/ephrin signaling to contractility regulation.

Finally, many studies interpret boundary-associated actomyosin enrichment as evidence for increased contractile tension. However, cytoskeletal remodeling encompasses multiple processes beyond contractility itself, including actin polymerization, filament turnover, and changes in cortical organization. Therefore, separating the effects of true contractile force generation from other cytoskeletal rearrangements remains an important experimental challenge. Addressing these limitations will require the integration of genetic, imaging, and quantitative biomechanical approaches to better define the mechanical role of Eph/ephrin signaling during tissue patterning.

Collectively, in this review, we summarized that Eph/ephrin signaling-driven actomyosin contractility is required for germ layer formation, hindbrain compartmentalization, eye field segregation, and cell segregation. To the best of our knowledge, this review will broaden researchers’ understandings of the mechanisms of cell sorting and boundary formation in the early embryonic development of vertebrates.

## Data Availability

No new data were created or analyzed in this study. Data sharing is not applicable to this article.
